# Prevalence of Café-au-Lait Spots in children with solid tumors

**DOI:** 10.1590/1678-4685-GMB-2015-0024

**Published:** 2016-05-24

**Authors:** Anna Claudia Evangelista dos Santos, Benjamin Heck, Beatriz De Camargo, Fernando Regla Vargas

**Affiliations:** 1 Departamento de Genética e Biologia Molecular, Universidade Federal do Estado do Rio de Janeiro (UNIRIO), Rio de Janeiro, RJ, Brazil; 2 Departamento de Genética, Universidade Federal do Rio de Janeiro (UFRJ), Rio de Janeiro, SP, Brazil; 3 Departamento de Pediatria, AC Camargo Cancer Center, São Paulo, SP, Brazil; 4 Departamento de Oncologia Pediátrica, Instituto Nacional de Cancer, Rio de Janeiro, RJ, Brazil; 5 Laboratório de Epidemiologia de Malformações Congênitas, Fundação Oswaldo Cruz, Rio de Janeiro, RJ, Brazil

**Keywords:** café-au-lait maculae, pediatric solid tumors, birth defects, nosology

## Abstract

Cafe-au-lait maculae (CALM) are frequently observed in humans, and usually are present as a solitary spot. Multiple CALMs are present in a smaller fraction of the population and are usually associated with other congenital anomalies as part of many syndromes. Most of these syndromes carry an increased risk of cancer development. Previous studies have indicated that minor congenital anomalies may be more prevalent in children with cancer. We investigated the prevalence of CALMs in two samples of Brazilian patients with childhood solid tumors, totaling 307 individuals. Additionally, 176 school children without diagnosis of cancer, or of a cancer predisposing syndrome, were investigated for the presence of CALMs. The prevalence of solitary CALM was similar in both study groups (18% and 19%) and also in the group of children without cancer. Multiple CALMs were more frequently observed in one of the study groups (Z = 2.1). However, when both groups were analyzed together, the significance disappeared (Z = 1.5). The additional morphological abnormalities in children with multiple CALMs were analyzed and compared to the findings observed in the literature. The nosologic entities associated with CALMs are reviewed.

## Introduction

Café-au-lait maculae (CALM) are named after their typical coffee-and-milk hue, and a color only slightly darker than the surrounding skin. There are two main types of CALMs. The most common type has fairly regular and clearly demarcated margins ("coast of California"). They may range in size from a few millimeters to several centimeters and may be present as solitary or as multiple spots. There is a second, less frequent type of CALM that has a much more irregular margin ("coast of Maine"), and is usually larger and solitary (Aase, 1990). At birth, a single CALM is observed in 5% of Caucasians and up to 15% of Americans of African descent. Three or more spots are observed in 2% of the population (Aase, 1990). CALMs may occur as isolated findings, or may be associated with minor and/or major congenital anomalies, as part of many syndromes. CALMs are typically observed in neurofibromatosis 1 (NF1), an autosomal dominant condition characterized by the presence of multiple neurofibromas, as well as other non-tumoral manifestations, like multiple CALMs, which are present in almost all adult patients with this disease. CALMs are usual findings in many other monogenic diseases, as well as in chromosomal abnormalities that are associated with mosaicism. This is the case with chromosomal rings, which are usually unstable during cell division, resulting in chromosomal mosaics. Interestingly, many of these genetically determined conditions that present with CALMs do also carry increased predisposition to cancer development. For this reason, we set out to investigate the prevalence of CALMs in children with and without cancer. Two groups of children with a pediatric solid tumor, resident in two states in southeastern Brazil (Rio de Janeiro and São Paulo), were independently examined for the presence of cutaneous pigmentary changes. A third group of children without cancer was also examined.

## Methods

Two study groups of children (ages 0-18 years) with current or previous diagnosis of a pediatric solid tumor were examined by a medical geneticist trained in dysmorphology. Study groups 1 and 2 are composed of children with current or previous diagnosis of a pediatric solid tumor: study group 1 (226 individuals) is from Rio de Janeiro (RJ); study group 2 (81 individuals) is from Sao Paulo (SP). The two cities are located in contiguous states in southeastern Brazil. Additionally, a third group (Study group 3) from Rio de Janeiro (RJ), and is composed of 176 school children without diagnosis of cancer or of a cancer predisposing syndrome. Age distribution among the three study groups did not show statistical differences. Both groups of children with cancer are part of a larger study aimed at investigating the prevalence of major and minor congenital anomalies in children with cancer. The same protocol for description of minor anomalies and morphologic variants based on the studies of Merks *et al.* (2003, 2006) was applied in all three study groups. The presence of two or more café-au-lait maculae was defined as "multiple CALMs". Analysis of the frequency of CALMs between the different study groups was accomplished through estimation of Z scores. We considered Z > 1.96 (two standard deviations) as the threshold for significance. Children with hematologic malignancies were not included in the study. Data on the size (diameter) of each individual café-au-lait spot, as well as ethnic background of the affected individuals were not collected in the present study. The present study was approved by the local Institutional Review Board of all involved institutions (83/08). Subjects were included in the study after discussion and signature of the informed consent by their parents and/or legal guardian.

## Results and discussion

In the present study, two study groups of patients with pediatric solid tumors were independently investigated for the presence of CALM by two trained dysmorphologists. The same protocol was applied in a third group of children without diagnosis of cancer or of a cancer predisposing syndrome.

Our study identified 28 children with multiple CALMs, 25 of which were associated with other congenital anomalies. The frequency of solitary CALMs was similar in the study groups 1 and 2 (children with solid tumors), and did not differ from that observed among school children without cancer (Z = 1.2 and Z = 0.5, respectively). The frequency of multiple CALMs, however, was higher in group 1 (12%) than in group 2 (5%), and also than that of the group of children without cancer (Z = 2.8). However, when both study groups 1 and 2 were analyzed together, the difference with the group without cancer became of borderline significance (Z = 2.0). These data are shown in Table 1. The additional findings observed in each patient are shown in Table 2. Table 3 shows the prevalence of each congenital anomaly within the patient group. A known syndromic diagnosis could be suspected in four patients. Additional patients presented multiple findings compatible with the clinical presumption of a dysmorphic, private syndrome (Table 2). Table 4 shows the main nosologic entities associated with multiple CALMs.

**Table 1:** Observed numbers of solitary and multiple CALMs in study groups 1, 2 and 3, along with expected numbers and Z scores for study groups 1, 2 and 1 + 2.

	cohort 1				
	N	total	%	E	Z
café-au-lait, solitary	40	200	20%	33	1.2
café-au-lait, multiple	24	200	12%	14	**2.8**
	cohort 2				
café-au-lait, solitary	15	81	19%	13	0.5
café-au-lait, multiple	4	81	5%	6	-0.6
	cohort 1 + 2				
café-au-lait, solitary	55	281	20%	46	1.3
café-au-lait, multiple	28	281	10%	19	**2.0**
	cohort 3 (control)				
café-au-lait, solitary	29	176	17%	-	**-**
café-au-lait, multiple	12	176	7%	-	**-**

Legend: E = expected number; N = observed number; Z = Z score. Statistically significant Z scores are marked in bold.

**Table 2:** Additional birth defects, congenital anomalies, and morphologic variants detected by ectoscopy in children with pediatric solid tumor and multiple CALMs. Cases are stratified by tumor and by cohort.

case	Tu	s	Age	additional congenital anomalies	syndr dx
study group 1					
1	Ccrc	f	10	obesity, macrocephaly, ocular hypertelorism	-
2	Cns	f	7	diastema	-
3	Cns	m	13	ephelides	NF1
4	Cns	m	6	broad halluces; hypo/depigmented spots; hemangioma	-
5	Ewi	f	18	upper limb asymmetry; thorax asymmetry; hyperconvex nails	-
6	Hb	m	1	epicanthus	-
7	Nb	f	17	-	-
8	Nb	f	4	epicanthus; anteverted nares; anti-Mongoloid palpebral slant; strabismus	-
9	Nb	f	3	clinodac; asymmetry; short, thin, brittle, hyperconvex nails; multiple nevi	-
10	Nb	f	15	partial syndactyly II-III; hyperconvex nails	-
11	Os	f	13	tall stature; obesity; macrocephaly; hypoplastic nails	-
12	Os	f	14	short stature; fetal pads; clinodactyly; multiple nevi	-
13	Pnsmt	m	10	hypo/depigmented spots; cleft lip/palate	?
14	Rb	m	5	-	-
15	Rms	f	14	hemangioma	-
16	Rms	m	10	anteverted nares	-
17	Rms	f	2	-	-
18	SS	m	15	partial syndactyly II-III; lower limb asymmetry; macrocephaly	-
19	Wt	m	3	transverse palmar crease; multiple nevi; partial syndactyly II-III feet	?
20	Wt	f	6	partial syndactyly II-III; hemangioma	-
21	Wt	m	7	macrostomia, lower limb asymmetry; fetal pads; multiple nevi, supernumerary nipple	SGB ?
22	Wt	m	7	genu varum; solitary nevus	-
23	Wt	m	9	multiple nevi	-
24	Sts	f	8	hallux valgus; haemangioma	-
study group 2					
25	Cns	m	12	prominent philtrum; low hanging columella; nevus of Ota; cow lick; thorax asymmetry	-
26	Fs	m	9	ocular hypertelorism, webbed neck, thorax asymmetry, winged scapulae, fibromata	JC ?
27	Wt	f	5	triangular face, frontal bossing, dry hair, prominent philtrum, high palate, camptodactyly	?
28	Gct	f	9	synophrys, heterochromia iris, microstomia, pectus carinatum, cubitus valgus, fibromata	NF1

Legend: age = age at examination (years); ccrc = clear-cell renal carcinoma; cns = central nervous system; ewi = Ewing sarcoma; fs = fibrosarcoma; gct = germ cell tumor; hb = hepatoblastoma; JC = Jaffe-Campanacci syndrome; nb = neuroblastma; NF1 = neurofibromatosis 1; os = osteosarcoma; rb = retinoblastoma; rms = rhabdomyosarcoma; s = sex; ss = synovial sarcoma; sts = soft tissue sarcoma; syndr dx = syndromic diagnosis; wt = Wilms tumor.

**Table 3:** Birth defects, congenital anomalies, and morphologic variants observed in children with pediatric solid tumor and multiple CALMs. Variants marked with an asterisk (*) have been previously associated with pediatric cancer (Merks *et al.*, 2008).

Additional congenital anomalies	N
growth	
tall stature *	1
short stature	2
thin, BMI < 20	2
overweight, BMI > 25	1
head / face	
macrocephaly *	3
Dolicocephaly	1
Brachycephaly	1
prominent forehead	1
cow lick	1
triangular face	3
round face	1
asymmetry, face	1
eye / ocular	
Hypotelorism	1
Strabismus	1
antimongoloid slant	1
epicanthal folds	5
Synophorus	1
heterochromia, Iris	1
almond shaped eyes	1
sparse eyebrows / eyelashes	5
long eyelashes	1
Ptosis	1
ear / auricular	
large earlobe / indentations earlobe	2
ear pit	1
Darwin's lump	1
Microtia	1
nose / nasal	
broad nasal base	2
flat nasal bridge	1
broad nose	2
broad nasal bridge	2
flat nose	1
low hanging columella	4
long / prominent philtrum	4
anteverted nares	5
hypoplastic alae nasae *	1
mouth / oral	
Macrostomia	1
cleft lip	1
Diastema	1
multiple frenulae	1
supernumerary tooth	1
torus palatinus	1
retro/micrognathia *	1
hypertrophy gum	1
hoarse voice	2
high arched palate	2
pointed chin	1
neck / trunk	
asymmetry, thorax	3
short / broad neck	2
pectus carinatum	1
hypertelorism, nipple	1
winged scapulae	1
scoliosis *	1
upper limbs	
asymmetry, upper limbs	1
Clinodactyly	5
Camptodactyly	1
cubitus valgus	4
transverse palmar crease	1
Sydney line *	3
ulnar deviation, fingers	1
fetal pads	3
hypoplastic / hyperconvex nails *	5
lower limbs	
asymmetry, lower limbs *	2
hypermobility, lower limbs	1
genu varum	1
curved tibiae	1
long feet	2
syndactyly II-III, feet	4
hallux valgus	2
skin / hair	
hemangioma *	4
Ephelides	1
nevus, solitary	3
nevus, multiple	5
hypo /depigmented areas	2
thin hair	1

**Table 4:** Main syndromes associated with CALMs.

	gene(s) / chromosomal regions	inher	CALMs	main neoplasias
monogenic (Mendelian)				
Ataxia-telangectasia^9^	*ATM*	AR	> 50%	leukemia, lymph, breast
Bloom^22,25^	*RECQL3*	AR	> 50%	leukemia, lymph, carcin
Cardio-Facio-Cutaneous^14,15,18^	*BRAF*, *MAP2K1*, *MAP2K2*, *KRAS*	AD	> 30%	ALL, HB, NHL
Carney complex	*PRKAR1A*	AD	< 50%	LCCSCT, PMS, endo tu
CMMRDS^16,27^	*MHS2*, *PMS2*, *MSH6*, *MLH1*	AR	> 50%	CRC, CNS, PST
Cowden^20^	*PTEN*	AD	< 50%	breast, thyroid, skin
Fanconi anemia^26^	*FANCA* to *FANCL*	AR/XL	< 50%	MDS, AML, solid tu
Gorlin^17^	*PTCH*	AD	< 50%	BCC, MB
Jaffe-Campanacci^23^	*NF1*	AD	> 99%	?
Legius^13,24^	*SPRED1*	AD	99%	-
Leopard^5^	*PTPN11*, *RAF1*, *BRAF*	AD	70%	NB
McCune-Albright^7^	*GNAS1* (somatic, mosaic)	spo	> 50%	sarcoma, breast, thyroid
MEN1^4^	*MEN1*	AD	40%	endocrine
Neurofibromatosis 1^13,24^	*NF1*	AD	> 95%	leukemia, GIST, breast
Neurofibromatosis 2^21^	*NF2*	AD	< 10%	CNS, schwannoma
Nijmegen^25^	*NBN*	AR	< 50%	lymph, solid tu
Noonan^15,18^	*PTPN11*, *SOS1*, *RAF1*, *KRAS*	AD	10%	JMML
RAPADILINO^19,22^	*RECQL4*	AR	< 50%	osteosarcoma
Rothmund-Thompson^11,22^	*RECQL4*	AR	< 50%	skin, osteosarcoma
Tuberous sclerosis^10,12^	*TSC1*, *TSC2*	AD	< 50%	CNS
Chromosomal				
Ring chromosome^8^	chromosomes 7,11,12,15,17,22	spo	> 50%	leukemia, WT
Chromosomal mosaicism^2^	mosaic trisomies / monosomies	spo	< 50%	leukemia
Uniparental disomy (UPD)				
Maternal UPD7^8^	7p	spo	< 50%	-
Russell-Silver^1,3,6^	11p15, 7p13	spo/AD	< 50%	WT, craniopharyngioma

Legend: AD = autosomal dominant; ALL = acute lymphoid leukemia; AML = acute myeloid leukemia; AR = autosomal recessive; BCC = basal cell carcinoma; carcin = carcinoma; CMMRDS = congenital mismatch repair deficiency syndrome; CNS = central nervous system; CRC = colorectal cancer; GIST = gastrointestinal stromal tumor; HB = hepatoblastoma; inher = inheritance; JMML = juvenile myelomonocytic leukemia; MB = medulloblastoma; MDS = myelodysplastic syndrome; MEN1 = multiple endocrine neoplasia 1; NB = neuroblastoma; NHL = non-Hodgkin lymphoma; spo = sporadic; WT Wilms tumor; XL = X-linked. References: 1 - Bruckheimer and Abrahamov, 1993; 2 - Carella *et al.*, 2010; 3 - Chitayat *et al.*, 1988; 4 - Darling *et al.*, 1997; 5 - Digilio *et al.*, 2006; 6 - Draznin *et al.*, 1980; 7 - Dumitrescu and Collins, 2008; 8 **-** Fujino *et al.*, 2010; 9 - Greenberger *et al.*, 2013; 10 - Józwiak *et al.*, 1998; 11 - Larizza *et al.*, 2010; 12 - Leung and Robson, 2007; 13 - Maertens *et al.*, 2007; 14 - Makita *et al.*, 2007; 15 - Ohtake *et al.*, 2011; 16 - Poley *et al.*, 2007; 17 - Ponti *et al.*, 2012; 18 - Rauen *et al.*, 2010; 19 - Sandoval *et al.*, 2012; 20 - Scheper *et al.*, 2006; 21 - Seminog and Goldacre *et al.*, 2013; 22 - Siitonen *et al.*, 2009; 23 - Stewart *et al.*, 2014; 24 - Wang *et al.*, 2012; 25 - Weemaes *et al.*, 1981; 26 - Giampietro *et al.*, 1993. 27 - Wimmer *et al.*, 2014.

Burwell *et al.* (1982) observed a single café-au-lait spot in 20% and multiple spots in 6% of 732 school children. Merks *et al.* (2006) observed 13% of single spots and 3.3% of multiple spots in healthy children. According to Tekin *et al.* (2001), single spots may be observed in up to 27% in children under 10 years of age.

Multiple café-au-lait spots were observed in cancer predisposing syndromes like neurofibromatosis 1, neurofibromatosis 2, tuberous sclerosis, McCune-Albright syndrome, Fanconi anemia, among others (Evans *et al.*, 1992; Giampietro *et al.*, 1993; Roach *et al.*, 1998; Kim *et al.*, 1999; Ferner *et al.*, 2007). More recently, café-au-lait spots were observed in 60% of carriers of biallelic mutations in mismatch repair (MMR) genes (Wimmer *et al.*, 2014), characteristic of constitutional mismatch repair deficiency syndrome (CMMRDS). These authors developed a score for investigation of this condition that includes the presence of CALMs and suggested that a score of three or more points warrants investigation of CMMRDS (Wimmer *et al.*, 2014).

Merks *et al.* (2005) studied 1,073 children with cancer and observed major abnormalities in 26.8% of the patients *vs.* 15.5% in controls (p = 0.001), and minor anomalies in 65.1% of the patients *vs.* 56.2% in controls (p = 0.001). Three or more minor anomalies were detected in 15.2% of the patients and in 8.3% of the controls (p = 0.001). A known cancer predisposing syndrome could be detected or suspected in 7.2% of the patients. The same authors (Merks *et al.*, 2008) observed 17 morphological abnormalities that occurred more frequently in pediatric cancer patients than in controls: blepharophimosis, asymmetric lower limbs, Sydney crease, broad foot, isolated short metacarpals, short distal phalanx of the thumb, portwine stain, hyperconvex nails, retrognathia, hypoplastic alae nasae, prominent ears, broad hand, scoliosis, hypertelorism, tall stature, macrocephaly, and microcephaly. Interestingly, CALMs are not included in this group. Two of these 17 congenital anomalies, blepharophimosis and asymmetric lower limbs, showed patterns of non-random association with other minor anomalies. The so-called "blepharophimosis pattern" consisted of the preferential association of blepharophimosis, increased anterior-posterior angulation of the spine, patchy hypopigmentation of the skin, and multiple CALMs. The asymmetric lower limb pattern consists of asymmetric lower limbs, tall stature, midface hypoplasia, ptosis, and pectus carinatum or excavatum.

It is worthy of note that, from 17 congenital anomalies described by Merks *et al.* (2008), eight were observed in at least one patient from our sample: tall stature, macrocephaly, ocular hypertelorism, hypoplastic alae nasae, retrognathia, scoliosis, hyperconvex nails, asymmetric lower limbs, and hemangioma. These anomalies are marked with an asterisk in Table 3. We did not observe any patient with the complete pattern described by Merks *et al.* (2008). However, two patients presented the association of multiple CALMS and hypopigmented / depigmented spots, suggestive of the "blepharophimosis pattern" (Table 3). It is important to take into consideration that we have included only patients with pediatric solid tumors in the study, while the sample studied by Merks *et al.* (2008) was composed of all pediatric malignancies. In fact, almost half (438 out of 1,073) of the children studied by Merks *et al.* (2008) were carriers of hematological malignancies (non-Hodgkin lymphoma, Hodgkin lymphoma, acute lymphoblastic leukemia, or acute myeloid leukemia).

In summary, our study showed that the frequency of solitary CALMs is not significantly increased in children with pediatric solid tumors. Multiple CALMs were more frequently observed in one of our studied samples (study group 1, Z = 2.8). However, the combined analysis of the studied samples (study groups 1 and 2) showed only borderline significance (Z = 2.0). Hence, the identification of multiple CALMs in a child with cancer warrants further morphologic evaluation, given the great number of cancer predisposing syndromes associated with multiple CALMs.
